# Quantitative Characterization of Cell Behaviors through Cell Cycle Progression via Automated Cell Tracking

**DOI:** 10.1371/journal.pone.0098762

**Published:** 2014-06-09

**Authors:** Yuliang Wang, Younkoo Jeong, Sissy M. Jhiang, Lianbo Yu, Chia-Hsiang Menq

**Affiliations:** 1 Precision Measurement and Control Laboratory, Department of Mechanical and Aerospace Engineering, The Ohio State University, Columbus, Ohio, United States of America; 2 The Ohio State University Comprehensive Cancer Center, The Ohio State University, Columbus, Ohio, United States of America; 3 Department of Physiology and Cell Biology, The Ohio State University, Columbus, Ohio, United States of America; 4 Center for Biostatistics, The Ohio State University, Columbus, Ohio, United States of America; Texas A&M University, United States of America

## Abstract

Cell behaviors are reflections of intracellular tension dynamics and play important roles in many cellular processes. In this study, temporal variations in cell geometry and cell motion through cell cycle progression were quantitatively characterized via automated cell tracking for MCF-10A non-transformed breast cells, MCF-7 non-invasive breast cancer cells, and MDA-MB-231 highly metastatic breast cancer cells. A new cell segmentation method, which combines the threshold method and our modified edge based active contour method, was applied to optimize cell boundary detection for all cells in the field-of-view. An automated cell-tracking program was implemented to conduct live cell tracking over 40 hours for the three cell lines. The cell boundary and location information was measured and aligned with cell cycle progression with constructed cell lineage trees. Cell behaviors were studied in terms of cell geometry and cell motion. For cell geometry, cell area and cell axis ratio were investigated. For cell motion, instantaneous migration speed, cell motion type, as well as cell motion range were analyzed. We applied a cell-based approach that allows us to examine and compare temporal variations of cell behavior along with cell cycle progression at a single cell level. Cell body geometry along with distribution of peripheral protrusion structures appears to be associated with cell motion features. Migration speed together with motion type and motion ranges are required to distinguish the three cell-lines examined. We found that cells dividing or overlapping vertically are unique features of cell malignancy for both MCF-7 and MDA-MB-231 cells, whereas abrupt changes in cell body geometry and cell motion during mitosis are unique to highly metastatic MDA-MB-231 cells. Taken together, our live cell tracking system serves as an invaluable tool to identify cell behaviors that are unique to malignant and/or highly metastatic breast cancer cells.

## Introduction

Cell behaviors, including morphology changes and migration variations, are reflections of intracellular tension dynamics. The study of cell behaviors is of significance in understanding many fundamental biological processes, such as wound healing [1], tissue repair [2], cell growth [3], chemotaxis [4] and immune responses [5]–[7]. Cell migration is a coordinated process with constant shape changes associated with assembly and disassembly of actin filaments from the leading edges to the trailing edges, respectively [8]. It plays an important role in embryonic development [9], during which, large amount of cells migrate collectively to form the three layer embryo. Stem cells then migrate from epithelial layers to target locations and differentiate to specialized cells that make up different tissues and organs [10]. Cell behaviors can also be related to the onset and progression of many diseases. For example, most cancer-related deaths are due to metastatic disease, which is a result of cancer cell migration from original locations to remote sites and the formation of secondary tumors [11]. Therefore, cell motility, which can be partially evaluated by cell instantaneous migration speed [12]–[17], is taken as an important factor that may correlate with the potential of cancer metastasis and invasion [16], [18]–[21].

Live cell tracking has been used to investigate and compare cell behaviors by measuring cell migration speed, monitoring migration trajectories, and examining temporal changes in cell shape and area [13], [15], [22], [23]. Automated cell tracking, however, suffers from various difficulties, such as the accuracy of cell lineage construction and simultaneous detection of cell boundaries during tracking. Most studies have, therefore, been limited to measuring instantaneous migration speed of the entire cell population [15], [16], [21]. Except for a few studies [22], the heterogeneity among cell behaviors has not been adequately addressed despite the well-recognized existence of heterogeneous subpopulations in established cell lines. Furthermore, the effects of different phases in cell cycle progression on cell behaviors cannot be addressed by employing a population-level approach. In this study, we aim to develop a live cell-tracking platform that allows us to conduct quantitative measurements of temporal changes in cell geometry and cell motion through distinct phases of the cell cycle for individual cells. We applied novel algorithms and necessary procedures to optimize cell imaging, cell segmentation, and separation of aggregated cells, along with off-line editing programs to further enhance accuracy of cell lineage construction and simultaneous detection of cell boundary over several cell cycles. Indeed, combination of automated segmentation and tracking with manual post-processing tools has been reported to be effective by others [22], [24], [25].

In general, cell tracking consists of three steps, cell imaging, cell segmentation, and cell association. Regarding cell imaging, fluorescence microscopic imaging [26] offers good image contrast. However, cells need to be either genetically engineered to generate fluorescent proteins or fluorescently labeled to enhance cell boundary information. Moreover, cells often suffer from photo bleaching that prevents frequent or long-term monitoring for live cell tracking. Bright field microscopic imaging can approximately estimate cell boundary by recording variations of light intensity at various vertical positions, as cells have greater variations in light intensity than the substrate [27]. Positive phase contrast microscopic imaging is also widely used in live cell tracking as cell bodies have lower light intensity than background [15], [23], [28]. However, mitotic cells and cells with increased cell height will show reversed image contrast, such that their cell bodies will have higher light intensity than background. In this study, we applied negative phase contrast microscope imaging to eliminate the confusion of image contrast reversion caused either by cell division or increased cell height. All cells including mitotic cells consistently show positive image contrast in our study.

Multiple algorithms have been applied for cell segmentation to extract information on cell location and cell boundary in acquired images. With spatial information of each cell location and the time interval among sequential cell images, one could determine migration speed and migration trajectory of any given cell being monitored. With cell boundary information, one could determine temporal variation and heterogeneity in cell geometry among monitored cells. The threshold method is a simple method that utilizes a threshold value to distinguish foreground and background areas in an image [29]. The threshold value is normally obtained through the overall light intensity distribution on cell bodies and substrates. This method highly depends on the image contrast of cells. Accordingly, cell segmentation is sensitive to the selection of the threshold value such that cells of low image contrast may not be detected. Another method for cell segmentation is region based active contour method [13], which can achieve robust cell segmentation and is less sensitive to quality of image contrast. However, this method suffers from difficulty in achieving optimized estimation of cell boundaries for individual cells due to its global based approach. In comparison, edge based active contour method, which utilizes the local light intensity information, can achieve optimized estimation of cell boundary [23]. The need of contour initialization for each target cell in this method makes it difficult to track all of the cells due to the presence of hundreds of cells in one field of view. In our live cell tracking system, cell segmentation was implemented by combining the “threshold method” for cell localization with the “modified edge based active contour method” for optimization of cell boundary detection. We refer to our modified edge based active contour method as “contour expansion method”. Consequently, boundary detection for all cells in the field of view is optimized and the capacity of massive cell tracking is not compromised.

Another challenge in cell segmentation is separation of aggregated cells. The watershed method has been applied to separate multiple cells in one detected area [30]. This method often suffers from over- or under-segmentation as it utilizes the shape of detected area to determine the number and corresponding boundary of individual cells by constructing Euclidian distance map within the detected area. In this study, we utilize cell light intensity information rather than shape of the detected area to improve cell separation. The geographic peaks of light intensity within the detected areas were used to determine the numbers and locations of multiple cells, and their corresponding boundaries were determined by a combination of threshold method with contour expansion method.

Cell association is a procedure that links cell identities and corresponding data for individual cells between any two consecutive frames [31]. The overlapping area based method, which assumes that the two segmented areas of the same cell in two consecutive video frames have the largest overlapping area, has been proven to be reliable [13]. The time interval between two consecutive video frames must be short enough to guarantee continuous tracking of the cells, in particular for cells with high migration speed. Cell mitotic detection is implemented by detecting one cell to two cells association between two consecutive frames. With the detection of cell division, cell lineage families can be constructed and a complete cell cycle can be defined as the duration between two events of cell division. Accordingly, temporal changes of cell geometry and cell motion along cell cycle progression in any given cell within the field of view can be determined.

Finally, we applied the cell-based approach, in which cell behavior parameters were associated with cell identities. This is in contrast to the widely used step-based approach that analyzes data obtained from all tracked cells between any two consecutive frames regardless of cell identity information [12]. The cell-based approach allows us to examine and compare temporal changes of cell behavior at single cell level. Therefore, the scope of heterogeneity in cell behaviors within an established cell-line can be studied. In addition to construct cell lineage families for all tracked cells, our live cell tracking program allows simultaneous measurement of cell shape, cell area, and cell migration features that reflect underlying intracellular tension dynamics of monitored cells. The capability of our live cell tracking system was evaluated using three well-characterized human breast epithelial cell lines, MCF-10A non-transformed breast cells, MCF-7 non-invasive breast cancer cells, and MDA-MB-231 highly metastatic breast cancer cells, with the objective to identify cell behaviors that are unique to malignant and/or highly metastatic breast cancer cells.

## Materials and Methods

### a. Cell culture

MCF-10A cells were maintained in 47.5% Dulbecco's modified Eagle's medium (DMEM) and 47.5% F-12 medium supplemented with 5% horse serum, EGF (20 ng/ml), bovine insulin (1 *µ*g/ml), hydrocortisone (0.5 *µ*g/ml), cholera toxin (0.1* µ*g/ml), NaHCO_3_ (0.2 mM), and 1% penicillin/streptomycin. MCF-7 cells and MDA-MB-231 cells were maintained in 90% RPMI 1640 medium supplemented with 10% fetal bovine serum and 1% penicillin/streptomycin. Cells were seeded in six-well plates for 24 hours, and were rinsed and replaced with fresh medium to remove debris that may interfere cell imaging. During imaging, cells were placed on a stage-top incubator (Model: WSKM-F1, Tokai Hit, Japan) with controlled humidity and temperature. The pH value of culture media was maintained by connecting the stage-top incubator with the pre-mixed air with 5% CO_2_ supplied through a CO_2_ controller (Model No.: DGTCO2BX, OKO Lab, Italy).

### b. Time-lapse negative phase contrast microscopy imaging

A phase contrast microscope (Model: IX51, Olympus) was used to record time-lapse image streams of the selected cell lines. A 10X negative phase contrast lens (Model: PLN10XPH/NH, Olympus) was used to get negative phase contrast images. A CCD camera (Model: C4742-95, Hamamatsu, Japan) was used to take images with 2 min intervals between two consecutive frames in a period of about 40 h, 70 h, and 75 h for MCF-10A, MCF-7, and MDA-MB-231 cells, respectively. Note that MCF-10A cells have a higher division rate, such that it almost achieves 100% confluence after 40 h monitoring. For each cell line, two independent experiments were conducted and had similar observation. We, therefore, only include data and analyses from one experimental trial.

### c. Development of automated cell tracking program

The implementation of automated cell tracking is briefly introduced below. It is divided into four phases, i.e., cell segmentation, cell association, off-line editing, and cell lineage construction.

#### c.1. Cell segmentation: contour expansion method and separation of aggregated cells

*Contour expansion method*: Cell segmentation includes cell localization and cell boundary detection. To automatically obtain cell locations and optimize cell boundary detection for all cells in the field of view, the threshold method is combined with a modified edge based active contour method in this study. The threshold method is applied to acquire cell localization and the initial contours of cell boundaries for all cells in the field of view in each video frame. The modified edge based active contour method is then applied to optimize the detection of cell boundaries based on local light intensity information for individual cells. With the traditional edge based active contour method, the contours are initialized outside the target objects. However, in this study, the initial contours are obtained from the masks generated by the threshold method and are gradually expanded driven by the field of gradient in light intensity. Accordingly, we refer to this method as the “contour expansion method”.

**Figure 1:**
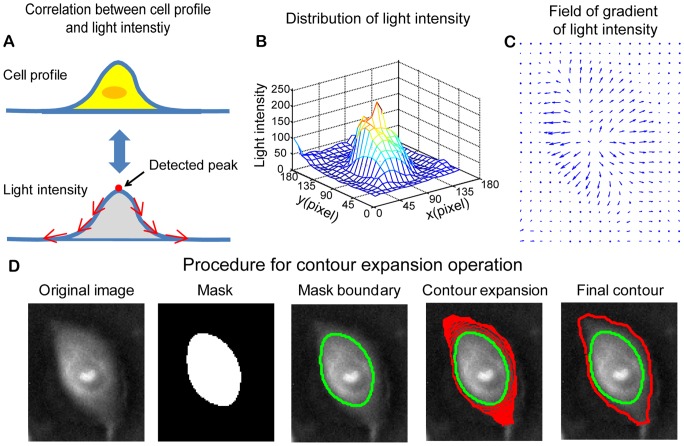
Contour expansion method for cell boundary detection. (A) It is assumed that most cells have illustrated height profiles with one peak located above the cell nucleus. In negative phase contrast images, the light intensity of cells is proportional to cell height. Therefore, the light intensity distribution over cell surface is similar to a height profile of cells with one peak located above each cell body. (B) Mesh plot of the light intensity for a selected cell (shown in **Fig. 1D**), which demonstrates the distribution of light intensity over cell surface. (C) Quiver plot of the gradient of light intensity for the selected cell. Over cell surface, the gradient of light intensity is pointing outwards. (D) Demonstration of contour expansion method for cell segmentation. The first figure shows the negative phase contrast image. The threshold method is used to get a preliminary mask for the selected cell, as shown in the second figure. The boundary of the mask is extracted and taken as the initial contour (the third figure). With contour expansion method, the initial contour is driven by the field of gradient of light intensity to gradually converge to the cell boundary (the fourth figure). The contour is finally converged at the boundary of the cell, where the contour achieves the minimum energy (the fifth figure).

For negative phase contrast cell images, light intensity is almost linearly related to the thickness of a phase object [32]. Normally, cells have a height profile as shown in the **top graph** of **Fig. 1A**. The apex of the profile locates above the cell nucleus. The height gradually decreases towards the cell boundaries. Therefore, one can expect that the light intensity over cell surface in negative phase contrast imaging would have similar profile with its height profile, as illustrated in the **bottom graph** of **Fig. 1A**.

**Fig. 1B** shows a mesh plot of the light intensity for a negative phase contrast cell image. One can see that light intensity has a higher value in the central area of the cell and then gradually decreases toward cell boundary area, which is consistent with the graph shown in **Fig. 1A**. One can obtain the field of gradient of light intensity by taking the differentiation of the light intensity along both *x* and *y* directions, as shown in **Fig. 1C**, which was used to define the outline of cell boundary in two-dimensional (2D) culture.

**Fig**. **1D** demonstrates the procedure of cell contour expansion. The **first figure** is the negative phase contrast image of a MCF-10A cell. A mask was obtained after applying the threshold method, as shown in the **second figure**. The boundary of the mask is extracted to serve as the initial contour, as shown in the **third figure**. The initial contour expands outward driven by the field of gradient of light intensity as indicated by the red contours shown in the **fourth figure**. The contour will stop at the cell boundary where it achieves the minimum energy. As shown in the **fifth figure**, the converged contour (red contour) had a much better approximation of the cell boundary than the initial contour (green contour). Note that the mask shown in the second figure can also be generated with the region based active contour method [13]. We tested both the region based active contour method and the threshold method and found no difference regarding the final result. Since the region based active contour method consumes more computation time, we applied threshold method to obtain masks in this study.

*Separation of aggregated cells:* As mentioned earlier, one major challenge in cell segmentation is separation of aggregated cells. Practically, a mask area initialized by the threshold method (or the region based active contour method) may contain more than one cell. Assuming the light intensity distribution across cell surface has the shape shown in **Fig. 1A**, one could determine the “number” and “location(s)” of cell(s) in one mask area by detecting the number of light intensity peaks in a given mask area.

**Figure 2:**
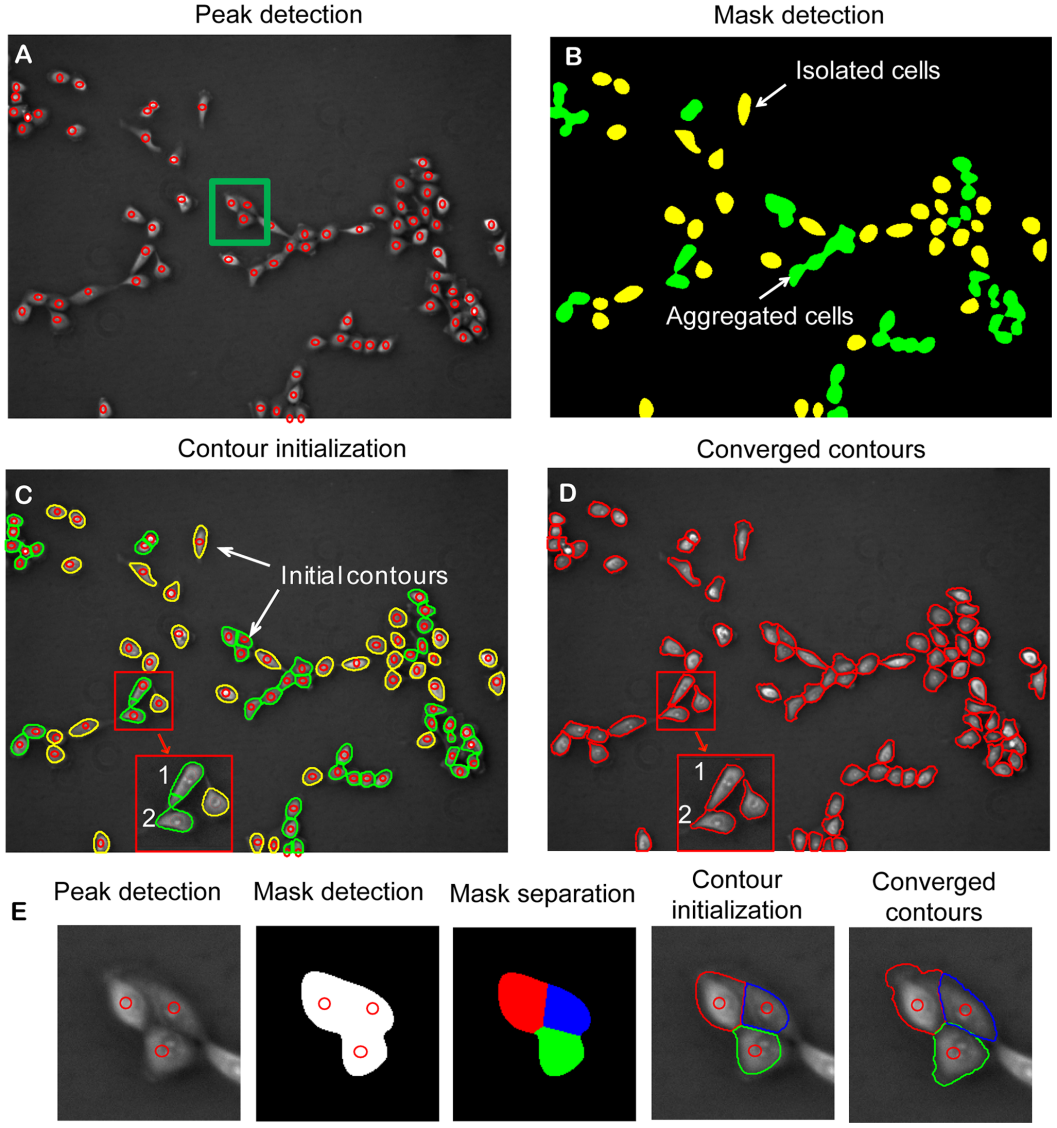
Cell localization, contour detection, and separation of aggregated cells. (A) Based on negative phase contrast image, peaks of light intensity are detected for all cells, as indicated by red circles. (B) Preliminary masks are obtained with the threshold method. Masks in green are areas with multiple peaks indicating aggregated cells. Masks in yellow are areas with single cell. (C) The boundaries of the preliminary masks are extracted to serve as initial contours for individual cells. (D) The contour expansion method is applied to detect cell boundaries for all cells in the field of view. (E) Steps taken to separate three aggregated cells selected in figure (A). The first figure shows three peaks detected, indicating three cells in the selected area. The second figure shows the mask area defined by the threshold method and locations of three detected peaks. The third figure shows division of the mask area into three sub-areas based on shortest distance between any given pixels and the three detected peaks. The fourth figure shows contour initiation by extracting the outlines of sub-areas. The fifth figure shows the final contours of three cells after contour expansion.

**Fig. 2** demonstrates the procedure of cell segmentation and separation of aggregated cells. **Fig. 2A** is a negative phase contrast image of MCF-10A cells with peaks detected for individual cells. Then the threshold method is applied to the image and a mask map is obtained, as shown in **Fig. 2B**, in which green masks are areas with multiple cells and yellow masks are areas with single cells. For mask with single cell, cell boundary is determined by contour expansion as described in **Fig. 1D**.

For mask with multiple cells, separation of the aggregated cells is demonstrated in **Fig. 2E**. The **first figure** shows that three cells are aggregated together with three detected peaks. After threshold operation, one mask area containing three peaks is obtained (the **second figure**). To operate contour expansion, initial contours must be determined for each cell within the mask area. As shown in the **third figure**, the mask area is separated into three subareas based on shortest distance between pixels and the three detected peaks. Each subarea is associated with one cell and the contour of the subarea is taken as initial contour for each cell (the **fourth figure**). By conducting contour expansion, the final contour for each involved cell is obtained as shown in the **fifth figure**.

After similar operations are performed to all masks with multiple cells, the initial contour for each cell in the field of view is determined (**Fig. 2C**). After contour expansion is performed for all cells in the field of view, the final segmentation result, shown in **Fig. 2D** **,** demonstrates the success in separating all aggregated cells. Note that error may occur during contour initialization. However, most errors are automatically corrected after contour expansion. As shown in the selected area in **Fig. 2C**, part of area in cell 1 is falsely assigned to cell 2. However, during contour convergence operation, the error was automatically corrected, as shown in the selected area in **Fig. 2D**.

**Figure 3:**
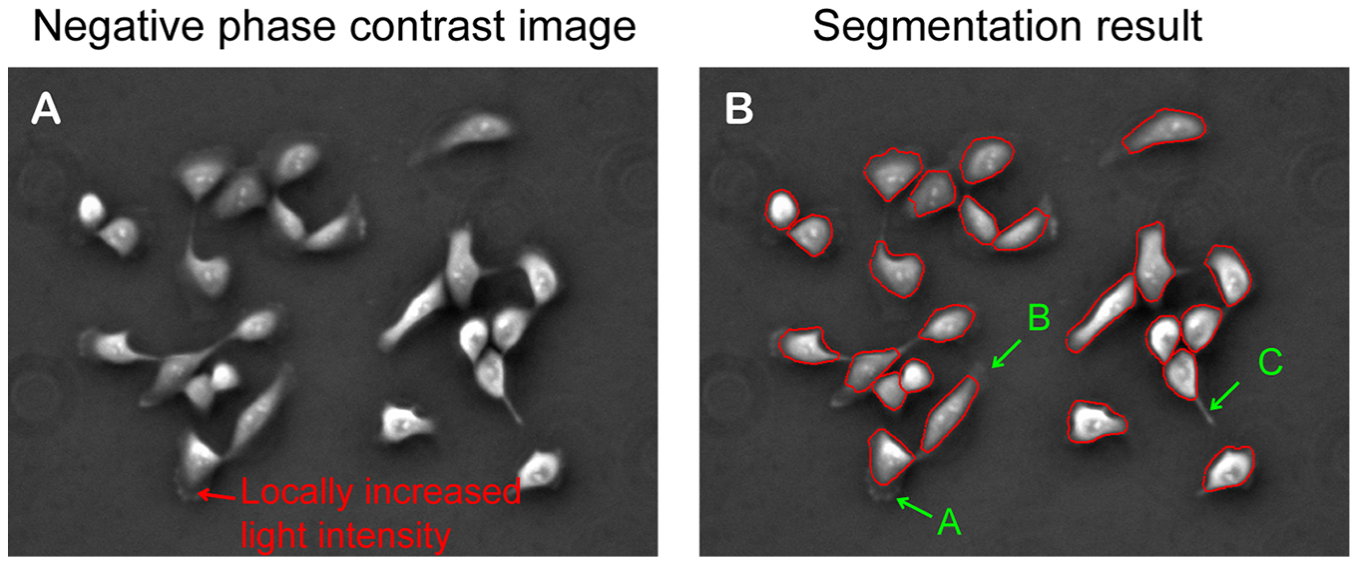
Peripheral protrusions may not be included in the final contours. The segmentation program can capture cell bodies and some parts of protrusion structures. However, peripheral protrusion structures with locally increased light intensity (marked by a red arrow in figure A) connected with thin extensions would not be included in the final contours (“A” in figure B). Similarly, thin lamellipodia of low image contrast would not be included in the final contours (“B” in figure B). Finally, the contour could not converge to a sharp protrusion structure due to the constraint of internal energy of the contour (“C” in figure B).

*Our contour expansion method captures cell body area and part of peripheral lamellipodia*: Compared to the corresponding negative phase contrast image shown in **Fig 3A**, the final contours of cell segmentation shown in **Fig 3B** demonstrated that our contour expansion method is quite effective in capturing most area of cell body for each individual cell. However, the final contours failed to capture part of the peripheral lamellipodia and filopodia, as marked in **A**, **B**, and **C** in **Fig. 3B**. During contour expansion, the contour stops at the positions with minimal light intensity and will not go further. Accordingly, protrusion structures with locally increased light intensity (marked by a red arrow in **Fig. 3A** **)** connected with thin extensions of cell periphery would not be included in the final contours (**A** in **Fig 3B** **)**. Similarly, thin lamellipodia of low image contrast would not be included in the final contours (**B** in **Fig 3B**). In both cases, contour convergence toward cell boundaries was terminated prematurely. Additionally, the contour could not converge to a sharp protrusion structure due to the constraint of internal energy of the contour (**C** in **Fig 3B**). Taken together, cell boundaries defined by the final contours reflect 2D geometry of each cell body with some portions of peripheral lamellipodia.

#### c.2. Cell association between two consecutive video frames and detection of cell division.

**Figure 4:**
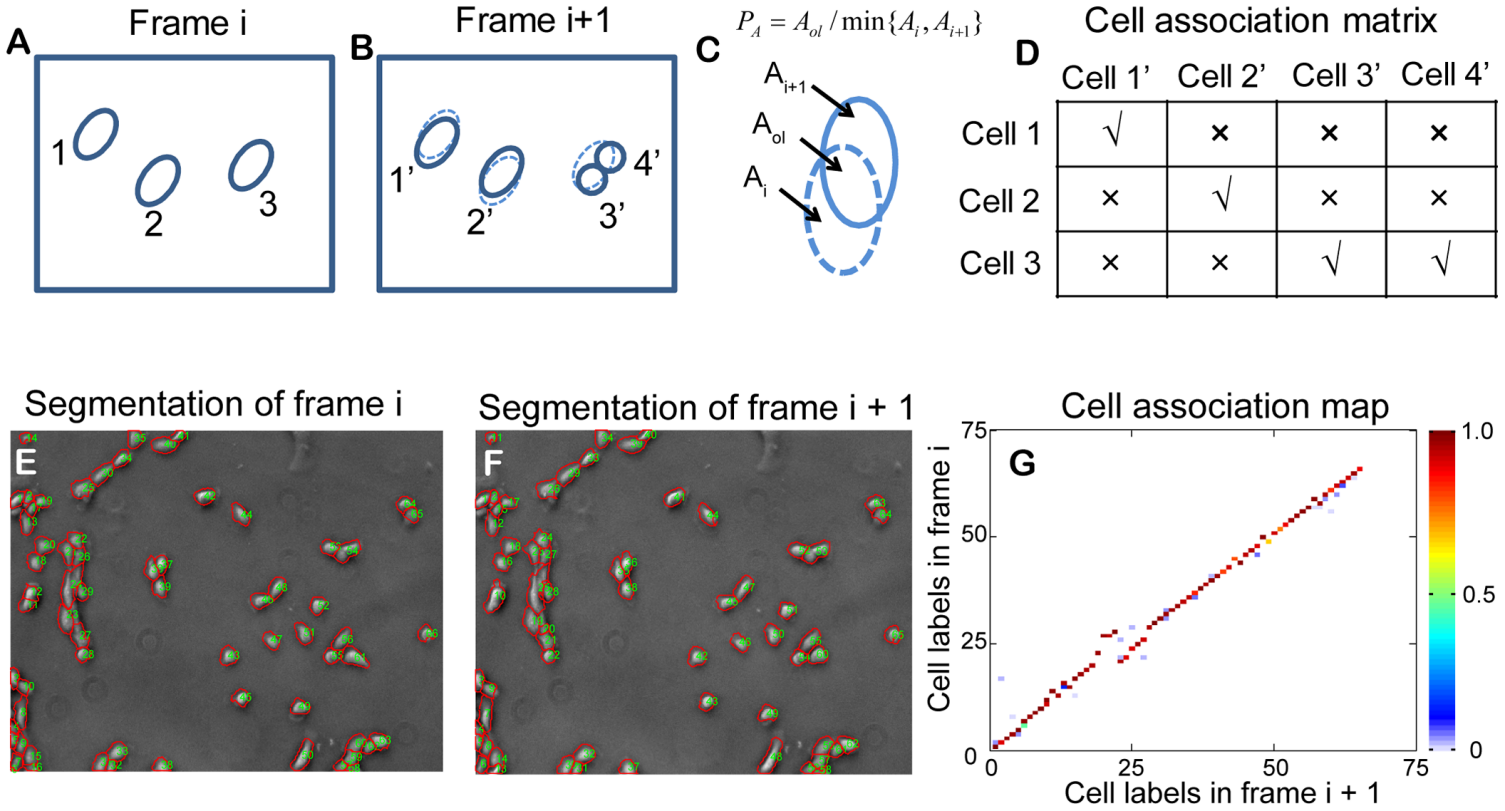
Cell association and detection of cell division. (A) Three detected cells in frame i. (B) Four detected cells in frame i+1. Cells 1′ and cell 2′ are the same cells of cell 1 and cell 2 in frame i, respectively. Cell 3 in frame i is divided into cell 3′ and 4′ in frame i+1. The cells can be associated based on the overlapping areas between the two consecutive frames. The two divided cells 3′ and 4′ in frame i+1 have large overlapping areas with their mother cell 3 in frame i. Moreover, the sum of the two areas has large overlapping area with the mother cell. This one-to-two association relationship is utilized to detect cell division. (C) The overlapping rate is defined as the ratio of the overlapping area to the minimum area of the two areas under investigation. (D) A cell association matrix is constructed with the calculated overlapping rate. (E), (F) Cell segmentation results for two consecutive frames. (G) The association matrix between two consecutive frames after calculating the overlapping rate. The *x* and *y* axes denote cell labels for cells in Frame i+1 and Frame i, respectively. The color of each point represents the overlapping rate (range from 0 to 1.0) of a given cell between two consecutive frames, as indicated by the color bar in the image.

Cell association, which links cells between any two consecutive video frames, is achieved by the overlapping area method. As illustrated in **Fig**.** 4A and 4B**, the mask of any given cell in a video frame has the largest overlapping area with its own mask in the subsequent video frame, provided that the time interval between two consecutive video frames is very short. In this study, the overlapping area is evaluated by overlapping rate *P*_A_, which is given as:


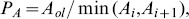
(1)

where *A*_i_ and *A*_i+1_ are mask areas in frame *i* and frame *i+*1, respectively, and *A*_ol_ is the overlapping area of *A*_i_ and *A*_i+1_ (**Fig. 4C**). For each detected cell in Frame *i*, the overlapping rate *P*_A_ will be calculated for masks within a selected area in Frame *i*+1. The one with the overlapping rate larger than a certain value will be linked to the cell.

For a cell undergoing division, both daughter cells in Frame *i*+1 will have a larger overlapping rate with their mother cell in Frame *i*. Meanwhile, the sum of the two areas in Frame *i*+1 also has large overlapping area with the cell in Frame *i*. This one-to-two association relationship is utilized to detect cell division. After calculating overlapping rates for all cells in Frame *i*, a cell association matrix can be generated, as illustrated in **Fig. 4D**. From the matrix, cell association and cell division can be determined. **Fig. 4E and 4F** show cell segmentation of Frame *i* and that of Frame *i*+1, respectively. The association matrix is constructed by calculating the overlapping rate for each possible pair of cells between two consecutive video frames. With this method, cells between two consecutive video frames are associated as shown in **Fig. 4G**.

**Figure 5:**
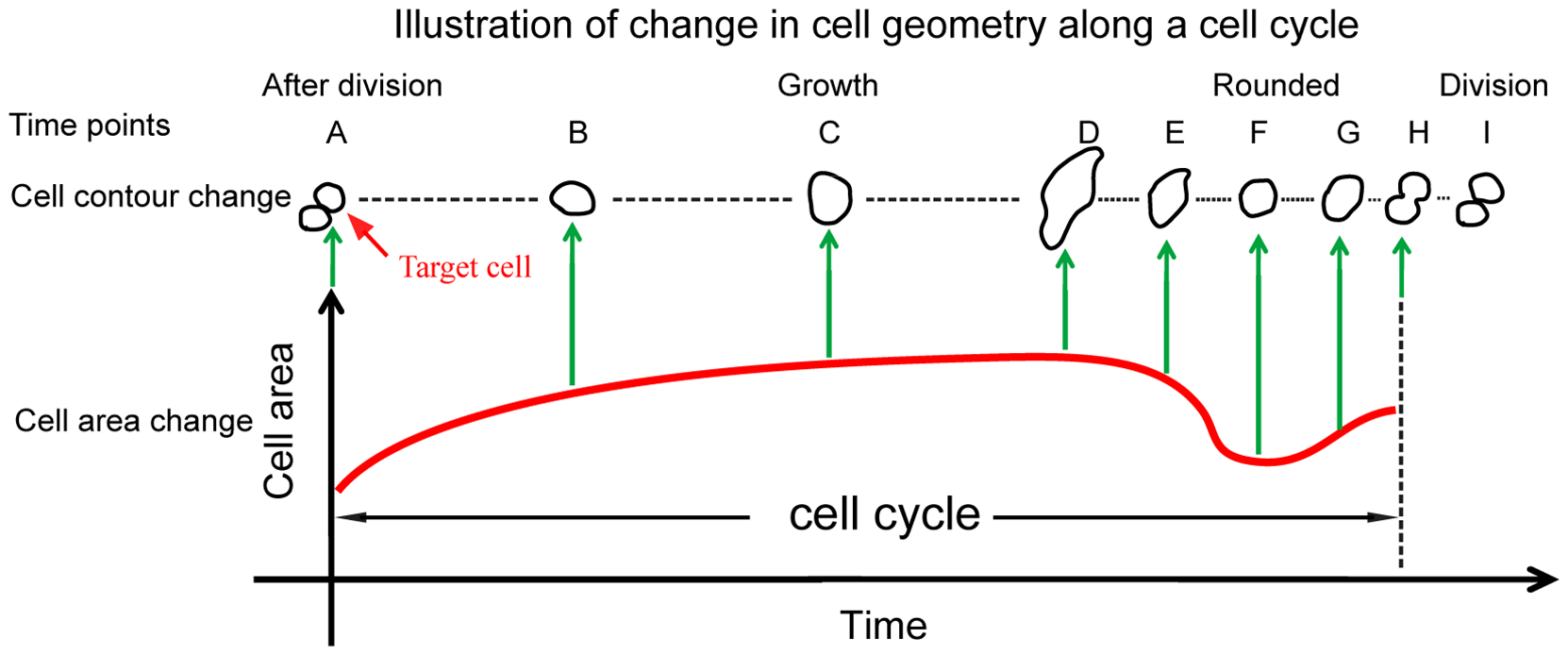
Illustration of temporal changes in cell area and cell shape along with cell cycle progression. After division, cell area gradually increases with change in cell shape from time point A to time point D. Thereafter, the cell becomes rounded with decreased cell area (time point F), marking the entry of mitotic stage. The cell is elongated with slightly increased area and subsequently a characteristic bottleneck structure appears and cell division occurs.

As shown in **Fig. 5**, when two cell bodies are formed which are detected by the emergence of two peaks of light intensity distribution in the mask area, the moment was defined as the completion of cell division and the beginning of the subsequent cell cycle (see the **time points H** and **A**). This moment is most likely to occur during late anaphase or early telophase of mitosis, as nuclear division precedes cytoplasmic division (cytokinesis). The duration of anaphase and telophase usually accounts for about 1% and 2%of the duration of cell cycle, respectively. Cell areas at 2D culture are usually the smallest at the beginning or by the end of cell cycle. When cells attach to the substrate, their 2D cell areas gradually increase with cell cycle progression (**time points B**, **C**, and **D**), a duration that corresponds to interphase of cell cycle that encompasses G_1_, S, and G_2_ phases. Thereafter, cells stop growth and become rounded-up to enter mitotic (M) phase. Mitosis accounts for approximately 10% of the duration of a cell cycle. Note that the cell axis ratio approaches one when cells become rounded up with decreasing cell area (**time point F**). Upon entering the anaphase, the cells are stretched into an oval shape with a slight increase in cell area (**time point G)**. The characteristic bottleneck structure appears indicating the progression of telophase (**time point H**), in which cytokinesis occurs at the same time once the nuclear envelop is reforming. In this study, the period from **time point A** to **time point H** is defined as an entire cell cycle. Note that once one-to-two cell association is detected, our automated cell-tracking program marked the completion of cell division and the beginning of the subsequent cell cycle.

#### c.3. Offline editing.

**Figure 6:**
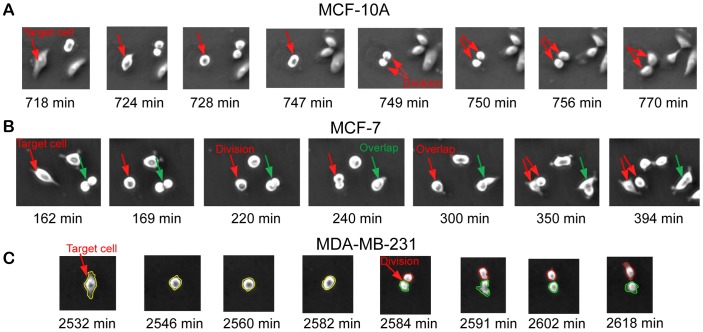
Cell behaviors during cell division for MCF-10A, MCF-7, and MDA-MB-231 cells. (A) MCF-10A cells divide horizontally with smooth separation after cell division. (B) Some MCF-7 cells divide vertically and remain vertically overlapping for a long time before the cell on the top slides down and gradually attaches to the substrate. (C) MDA-MB-231 cells are unique in that divided cells often underwent rapid motion and irregular geometry change, as shown with green contours from the fifth to the seventh figures.

During automated cell tracking, low image contrast and cells moving out the field of view can result in loss of tracking. Additionally, false detection of cell division is one of the leading error sources that contribute to false cell tracking. Differences in cell behaviors during cell division among MCF-10A, MCF-7, and MDA-MB-231 cells were observed. For MCF-10A cells, all cells divided horizontally and the two daughter cells move away in opposite direction from the original location of the mother cells (**Fig. 6A** **).** The separation process between two daughter cells occurs smoothly. Hence, cell division for MCF-10A cells can be detected at a relative high accuracy. For MCF-7 cells, some cells divided vertically and remained vertically overlapped for some time before the cell on the top sliding down and gradually attached to the substrate (**red arrows** in the fourth figure of **Fig. 6B**). In some MCF-7 cells, horizontal divided cells became vertically overlapping before their attachment on substrate (**green arrows** in the fourth figure of **Fig. 6B** **)**. Accordingly, the time point of the occurrence of cell division was delayed and the false two-to-one cell association was interpreted as loss of tracking, respectively. The challenges seen in MCF-7 cells also occur in MDA-MB-231 cells. MDA-MB-231 cells are unique in that divided cells often underwent rapid motion and irregular geometry change as shown with **green contours** in **Fig 6C**. Furthermore, MDA-MB-231 cells often “dance” around with their adjacent cells that lead to abrupt interchange of spatial locations among interacting cells. The abrupt motion due to rapid contraction of focal adhesions can lead to loss of tracking. Taken together, rapid and dynamic MDA-MB-231 cell behaviors represent the utmost challenge in live cell tracking.

To construct cell lineage trees, only cells with their offspring cells remaining in the field of view were selected for analysis. To identify and correct false cell tracking for all monitored cells, a program was developed to display tracking trajectories of specified cells frame by frame. The program allows us to manually but effectively check the accuracy of cell tracking for each individual cell. The occurrence of false cell-association and false loss-of-cell-tracking as well as their corresponding frame numbers are identified and recorded for subsequent off-line editing. For cells with false loss-of-tracking, the offline-editing program links the same cell and reconstructs the database. For cells with false cell-association, the program relinks the correct cells and reconstructs the database. For cells divided vertically and remained vertically overlapped, the exact time when cell division occurred can not be certain even after off-line editing. Thus, cells overlapped vertically for extensive period of time were abandoned for further analysis. By doing so, all the data included for analysis in this study are of high accuracy.

After off-line editing, only cells remaining in the field-of-view and without uncorrectable false tracking are included for data analysis. The total number of cells analyzed includes selected cells in the first generation as well as their offspring cells from different generations. The performance of our automated cell-tracking program was evaluated and summarized in **Table 1**. The false tracking rates over all tracking events for selected MCF-10A, MCF-7, and MDA-MB-231 cells are 0.14%, 0.04%, and 0.10%, respectively. All MCF-10A cells were selected for analysis as all false tracking events could be corrected easily. However, one cell lineage of MCF-7 cells and three cell lineages of MDA-MB-231 cells were excluded for analysis due to their uncorrectable false tracking events. Moreover, false tracking events of MCF-10A cells increased considerably at high cell density due to its higher proliferation rate (**Fig. S1**). At comparable cell density, the overall false tracking rate of MCF-10A cells was similar to MCF-7 cells, and was lower than MDA-MB-231 cells. MCF-10A cell-tracking movies before and after off-line editing are included in (see **Video S1**, **Video S2**, and **Video S3**).

**Table 1:** False tracking rate of selected MCF-10A, MCF-7, and MDA-MB-231 cells.

Cell lines	Total tracking event a	False association event ^b^	Loss of tracking event ^c^	Overall false tracking rate
**MCF-10A**	73672	95 (0.13%)	8 (0.01%)	103 (0.14%)
**MCF-7**	39795	13 (0.03%)	4(0.01%)	17 (0.04%)
**MDA-MB-231**	28520	22 (0.08%)	7(0.02%)	29 (0.10%)

a Total tracking event is the sum of the all cell-tracking events over the total selected cells that underwent an entire cell cycle. **^b^**False association is mostly due to false detection of cell division, rapid change of cell locations among multiple interacting cells. **^c^**Lost tracking is mainly due to low image contrast, abrupt motion due to rapid contraction of focal adhesion. Note that cells moving out the field-of-view were not counted as false tracking events.

#### c.4. Cell lineage construction with quantitative measurement of change in cell behavior along cell cycle progression

With correct cell segmentation, cell association, and detection of cell division, cell behavior information such as cell body geometry and cell motion can be readily extracted and aligned with cell cycle progression. Cell area was obtained by calculating the calibrated pixel size in the area enclosed by the final contour of a given cell. In this study, cell shape was evaluated by cell axis ratio. For axis ratio measurement, the detected cell contours were mathematically fitted as ellipses, and the ratio of long axis to short axis of the fitted ellipses is defined as the axis ratio. During cell tracking, the centroids of the detected cell contours were taken as cell locations and to construct cell migration trajectories. Based on the cell centroids, the displacement between any two successive frames for a cell can be determined. The instantaneous migration speed can then be calculated by dividing the displacement of a cell between two consecutive frames by the time interval of the frames, i.e. 2 min, in this study.

**Figure 7:**
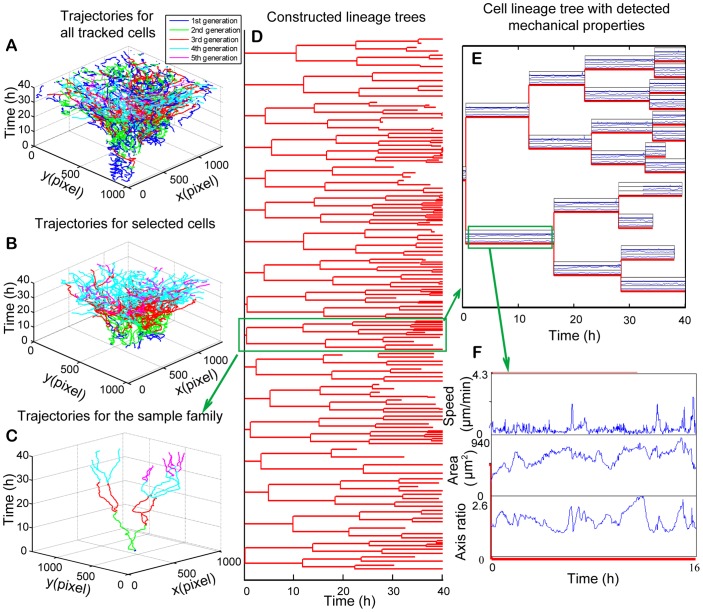
Cell lineage construction and associated cell behaviors after offline editing. (A) Cell trajectories for all tracked cells in the-field-of-view. The color of trajectories indicates cell generations. Blue, green, red, cyan, and magenta represent trajectories from the first to the fifth generations, respectively. (B) Cell trajectories for cells presented in the first video frame and their offspring cells. (C) Trajectories for cells from a selected cell lineage family. (D) Cell lineage families constructed with automated cell tracking program followed by offline editing. (E) A selected cell lineage family with cell migration speed, cell area, and cell axis ratio aligned with cell cycle progression. Note that a maximum of 5 generations and 11 cell divisions in total are observed in this family during 40 hrs of monitoring. (F) The enlarged figure for the selected area in figure (E).

In addition to quantitative measurement of temporal change in cell behaviors, cell lineage for all tracked cells can be constructed. **Fig. 7** shows the cell-tracking result of MCF-10A cells during 40 h monitoring. **Fig. 7A** shows cell trajectories for all the cells in the-field-of-view during cell tracking, including cells moving in or moving out of the field-of-view during the experiment. Different colors indicate different generations. **Fig. 7B** shows the trajectories of the original cells present in the first video frame and their offspring cells that stayed in the field-of-view after correction of false tracking with the off-line editing program. Those cells moving into the-field-of-view at later time are excluded. The corrected cell lineage families are shown in **Fig. 7D**. One cell lineage family was selected (marked with a **green box**) and the trajectories of 23 cells in the family are shown in **Fig. 7C**. From the corresponding cell lineage tree shown in **Fig 7E**, there are 11 divisions detected and totally five generations in this selected family within 40 h of monitoring. Cell behavior parameters were aligned with cell lineage trees. **Fig. 7F**, which showed the synchronized plot of instantaneous migration speed, cell area, and cell axis ratio along a complete cell cycle, is an enlarged plot of the selected area in **Fig. 7E**. Taken together, our live cell tracking system allows us to examine and correlate cell area, cell axis ratio, and cell migration speed along with cell cycle progression at single cell level within the context of its cell lineage.

### d. Determination of cell motion type

The theory of particle movement was applied to further characterize cell migration [12]. Based on motion trajectory, mean squared displacement (MSD) can be calculated and be used to differentiate cell motion type [33], [34]. MSD is a measure of the average distance a cell travels over a specified time interval, *τ*. Since cell motion trajectory is recorded at a constant sampling time, *δt*, the time interval is specified as an integer multiple of *δt*, i.e., *τ = nδt*. Therefore, when considering a cell motion trajectory having *N* data points (numbered from 1 to *N*), the number of data pairs separated by the specified time interval is *N − n*. The MSD over all data pairs separated by the specified time interval is given as


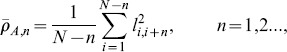
(2)

where *l_i,i+n_* is the displacement between the two data points, i.e., point *i* and point *i+n*.

**Figure 8:**
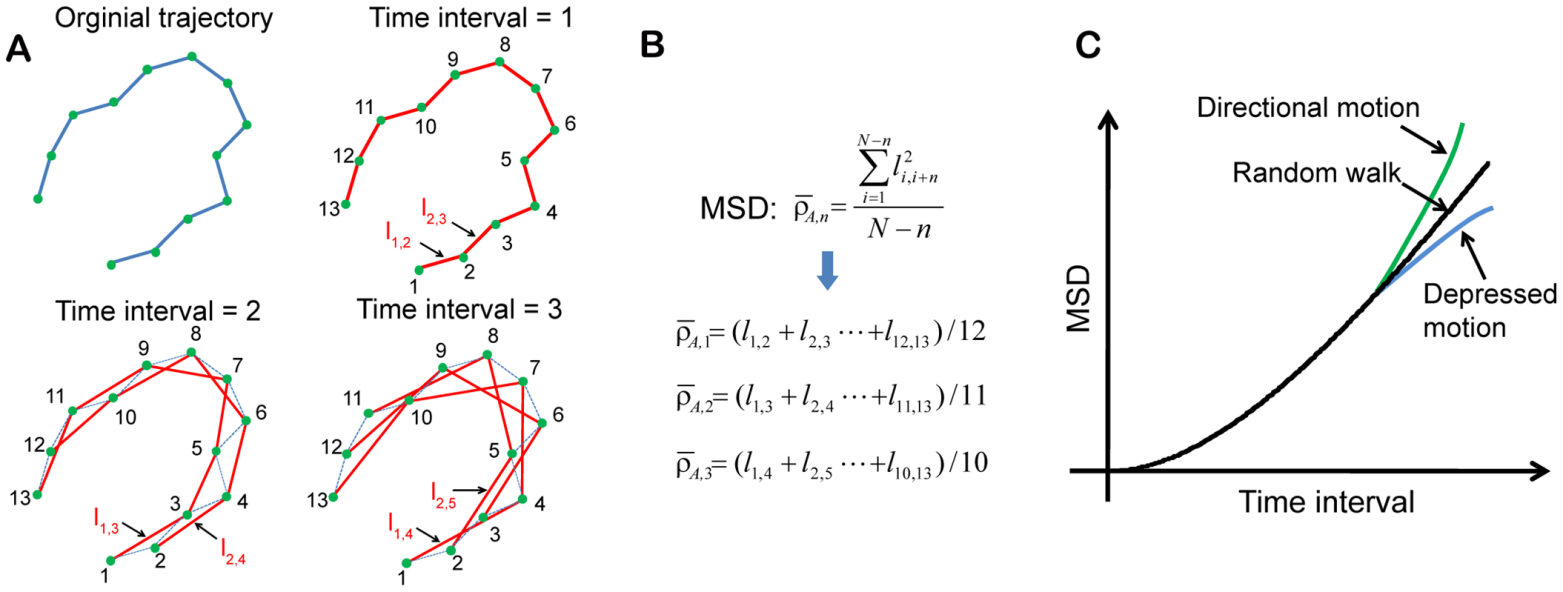
Calculation of mean square displacement (MSD) and determination of motion type. (A) Schematic showing the selected pairs of data point along a trajectory with different time interval for the calculation of MSD. (B) MSD calculation. (C) MSD as function of time interval to determine motion type. The linear increase of the MSD with the time interval indicates the motion type of random walk. Directional motion leads to a MSD curve deflected upward, whereas depressed motion results in a MSD curve deflected downward.

**Fig. 8** illustrates MSD calculation and motion type determination. Since the number of data pairs decreases as the specified time interval (*τ = nδt*) increases, the uncertainty of the MSD calculation associated with large *n* increases. Therefore, *n* is usually limited to below one-quarter of the total number of data points [34]. For the trajectory of 13 data points shown in **Fig. 8A**, the maximum *n* was set to be 3. As shown in **Fig. 8B**, only three MSDs are to be calculated. **Fig. 8C** illustrates three MSD curves versus the time interval, *τ*, which are indicative of three distinct motion types. Each time interval in the figure is integer multiples of the sampling time, which is dictated by the frame rate specified for image capturing during cell monitoring. The linear increase of the MSD with the time interval indicates the motion type of random walk. Directional motion leads to a MSD curve deflected upward, whereas depressed motion results in a MSD curve deflected downward.

### e. Statistical analysis

Median values of multiple measurements of cell area, cell axis ratio, cell instantaneous migration speed, and cell migration range from each single cell that underwent an entire cell cycle were calculated. Nonparametric Wilcoxon rank sum test was then applied to test distribution shift of these median values among cell lines investigated (MCF-10A, MCF-7, and MDA-MB-231). The *p* values of all 12 comparisons were adjusted by Holm's method. If the adjusted *p* value of a particular comparison is smaller than the significance level 0.05, one can conclude that two cell lines are different for the feature tested. Fisher's exact test was used to compare differences in motion types for MCF-10A, MCF-7, and MDA-MB-231 cells that underwent an entire cell cycle.

## Results and Discussion

The capability of our live cell tracking system is applied to three well-characterized human breast cell lines, MCF-10A, MCF-7 and MDA-MB-231. In addition to investigate variations in cell body geometry and cell motion for monitored cells, we also conduct quantitative measurements of temporal change in cell behaviors along cell cycle progression for cells that underwent an entire cell cycle. For cell geometry, cell area and cell axis ratio were studied. For cell motion, instantaneous migration speed, motion type, motion range, and migration direction related to the long axis of cells were analyzed.

### a. Cell geometry

#### a.1. Two-dimensional geometry distribution map

**Figure 9:**
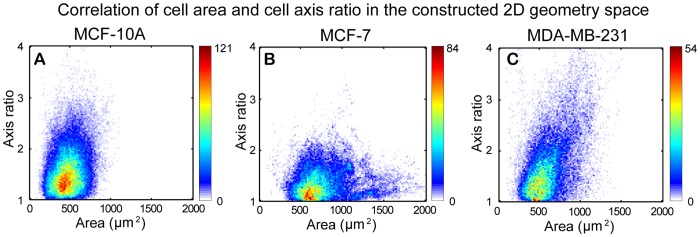
Correlation between cell area and cell axis ratio in a constructed 2D geometry space. For any point in the 2D space, the *x* coordinate and the *y* coordinate represent cell area and cell axis ratio of a given cell at each video frame, respectively. Summation of all entry data in the 2D space for all video frames results in the overall 2D geometry distribution maps. The frequency of cells appears at each point in the 2D space is indicated with corresponding color bar. (A) MCF-10A cells have a narrow distribution of cell area, while (B) MCF-7 cells show a wider distribution of cell area. (C) MDA-MB-231 cells have a wider distribution of axis ratio. Moreover, the axis ratio for MDA-MB-231 cells slightly increases with increasing cell areas.

To examine the correlation between cell area and cell axis ratio for each cell-line, a 2D geometry distribution map was constructed. The value of cell area *A* and axis ratio *R* for each given cell in each video frame are integrated as a single point [*A*, *R*] in the 2D space. The [*A, R*]s for all selected cells in each video frame are displayed in the 2D space. Summation of all video frames results in the overall distribution of cell area and axis ratio for all tracked cells, which we refer to as 2D geometry distribution map. The 2D geometry distribution maps for MCF-10A, MCF-7, and MDA-MB-231 cells are shown in **Fig. 9A, 9B, and 9C**, respectively.

MCF-10A cells had a relative narrow distribution in cell area. Note that cells with higher axis ratios did not appear to have larger cell areas. MCF-7 cells have the widest distribution in cell area yet had the narrowest distribution of axis ratio, indicating that most MCF-7 cells had round cell body geometry yet with various sizes of cell area. MDA-MB-231 cells had the widest distribution of axis ratio and relative wide distribution of cell area. Moreover, for cells with increased axis ratio, the cell area tends to increase as well. MCF-10A and MDA-MB-231 cells have similar cell area distribution and cell axis ratio distribution, however their 2D geometry distribution map appeared to be quite different. The axis ratio of cell body for MCF-10A cells was less dependent on the change of cell area, whereas the axis ratio of cell body in MDA-MB-231 cells increased with increased sizes of cell area. In general, MDA-MB-231 cells had a greater extent of heterogeneity in cell shape compared to MCF-10A and MCF-7 cells.

#### a.2. Cell geometry and cell cycles

**Figure 10:**
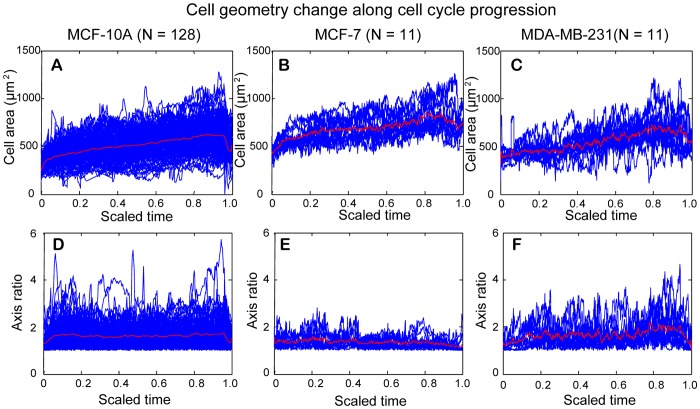
Temporal change of cell area and cell axis ratio along cell cycle progression for MCF-10A, MCF-7, and MDA-MB-231 cells. The cell cycle was scaled to 0–1 to facilitate comparison among different cells within the same cell-line. Values of cell area or cell axis ratio for each cell examined (blue curves) were lined up with the scaled cell cycle and the mean value for each parameter was shown by the red curve in each figure. (A–C) A rapid increase in mean size of cell area occurred shortly after cell division, which reflects cell attachment on the substrate after cell division. Thereafter, mean size of cell area gradually increased. A rapid decrease in mean size of cell area occurred before the end of cell cycle, which reflects cells becoming rounded in preparation of cell division. (D–F) The mean axis ratio was lowest before and after cell division for the three cell lines, reflecting cells rounded up before and after cell division. Otherwise, the mean axis ratio did not change much at the interphase of cell cycle for both MCF-10A cells and MCF-7 cells. In comparison, the mean axis ratio for MDA-MB-231 cells slightly increased with cell cycle progression.

The construction of cell lineage as well as the measurement of cell behaviors along cell cycle progression makes it possible to study temporal changes in cell geometry along cell cycle progression. To compare temporal changes of cell behaviors along cell cycle progression among individual cells that underwent the entire cell cycle, cells with different cell cycle lengths are normalized to the same scale ranging from 0 to 1. The quantitative values of cell behaviors were then lined up with the scaled cell cycle for each cell. **Fig. 10A-C and 10D-F** show the temporal change of cell area and cell axis ratio along progression of cell cycle in the three cell lines, respectively. In each figure, the blue curves are collective data for individual cells that underwent an entire cell cycle in each cell line. The red curve is the mean value of collective data, which offers to evaluate the overall trend of cell behavior along with cell cycle progression for each cell line. The extent and the occurrence of heterogeneity among individual cells from the mean value along cell cycle progression could be assessed in the plots generated.

**Fig. 10A-C** shows the change of the cell area along the scaled cell cycle for the three cell lines. A rapid increase in mean size of cell area occurred shortly after cell division, which reflects cell attachment on the substrate after cell division. Thereafter, mean size of cell area gradually increased. A rapid decrease in mean size of cell area occurred before the end of cell cycle, which reflects cells becoming rounded in preparation of cell division. Shortly after, cell area slightly increased, reflecting the cells were entering anaphase and telophase before cytokinesis (see **Fig. 5** for corresponding time points). Note that most cell division detection in our program occurs briefly before the actual event of cytokinesis, as detection of two daughter cells is dictated by the emerging of two cell bodies rather than their separation.

**Fig. 10D-F** shows the change of axis ratio along the scaled cell cycle for the three cell lines. The mean axis ratio was lowest before and after cell division for all cell lines, reflecting cells rounded up before and after cell division. Otherwise, the mean axis ratio did not change much at the interphase of cell cycle for both MCF-10A cells and MCF-7 cells. In comparison, the mean axis ratio for MDA-MB-231 cells slightly increased with cell cycle progression. Taken together, most cells become rounded up before mitosis, which is consistent with our data showing that the smallest size of cell area with lowest axis ratio occurred at the end or at the beginning of cell cycle.

**Figure 11:**
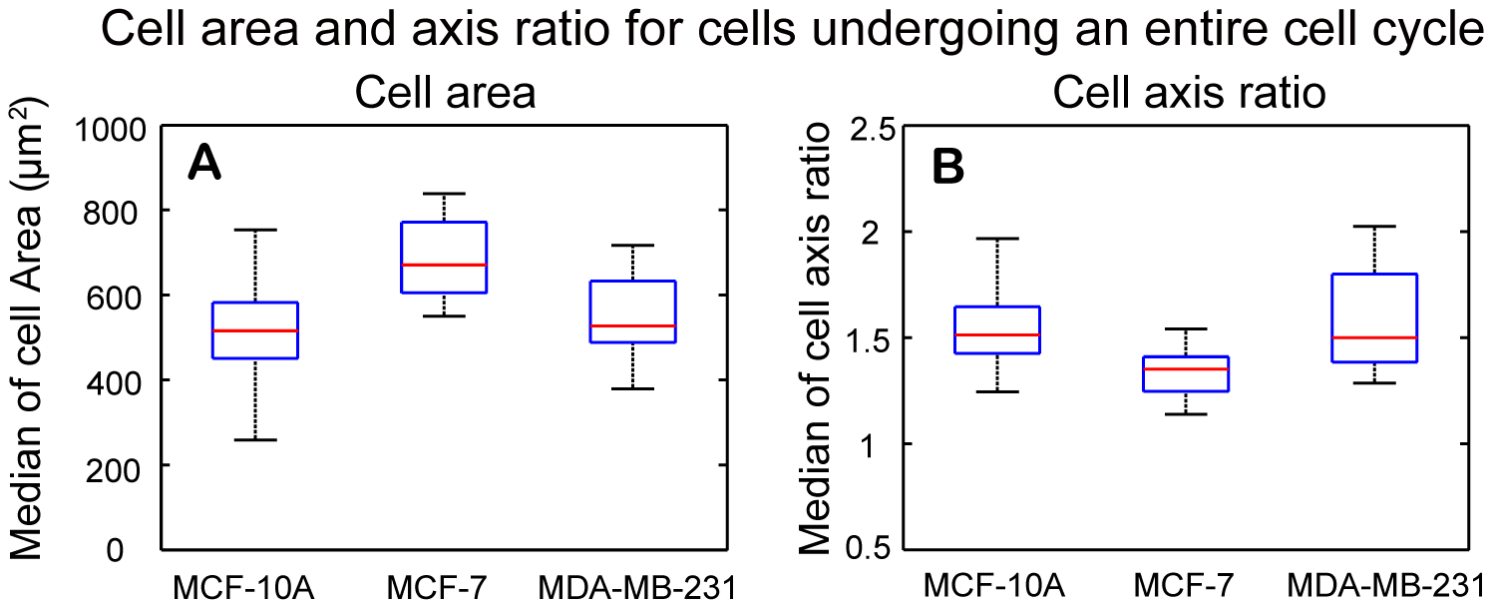
Boxplots of median cell area and median cell axis ratio for cells undergoing an entire cell cycle. (A) MCF-7 cells have highest median value of cell area, while the median value for MDA-MB-231 cells is slightly larger than that for MCF-10A cells. The Wilcoxon rank sum test showed that there is statistically significant difference in distribution shift of median cell areas between MCF-10A and MCF-7, and between MDA-MB-231 and MCF-7 cells. (B) MDA-MB-231 cells have the highest median value of cell axis ratio followed by MCF-10A cells. MCF-7 cells have the lowest median value of cell axis ratio among the three cell lines. The Wilcoxon rank rum test showed that there is statistically significant difference in distribution shift of median cell axis ratios between MCF-10A and MCF-7, and between MDA-MB-231 and MCF-7 cells.

The median values of cell area and cell axis ratio for each cell that underwent an entire cell cycle were determined and statistical analysis was applied to test the distribution shift of the median values of cell area and axis ratio among the three cell lines. **Fig. 11A** shows the boxplots of median cell areas for MCF-10A, MCF-7, MDA-MB-231 cell replicates. Among the three cell-lines, MCF-7 cells had the largest median cell area of 670.7 µm^2^. The median cell areas of MCF-10A cells and MDA-MB-231 cells are 515.8 µm^2^ and 527.3 µm^2^, respectively. Nonparametric Wilcoxon rank sum test showed a statistically significant difference in distribution shift of median cell areas between MCF-10A and MCF-7 cells, and between MDA-MB-231 and MCF-7 cells (**Table 2**). **Fig. 11B** shows the boxplots of median cell axis ratios for MCF-10A, MCF-7, MDA-MB-231 cell replicates. The median cell axis ratios are 1.51, 1.35, and 1.50 for MCF-10A, MCF-7, and MDA-MB-231 cells, respectively. Nonparametric Wilcoxon rank sum test indicated a statistically significant difference in distribution shift of median cell axis ratios between MCF-10A and MCF-7 cells, and between MDA-MB-231 and MCF-7 cells (**Table 2**).

**Table 2:** Unadjusted *p*-values of Wilcoxon rank sum test.

Comparison group	Cell area	Cell axis ratio	Migration speed	Motion range
MCF-10A vs. MCF-7	1.181×10^−5^	8.275×10^−5^	0.1522	6.549×10^−8^
MCF-10A vs. MDA-MB-231	0.4016	0.8606	2.008×10^−5^	0.8976
MDA-MB-231 vs. MCF-7	0.004102	0.00833	0.006634	2.835×10^−6^

### b. Cell motion

#### b.1. Instantaneous migration speed

**Figure 12:**
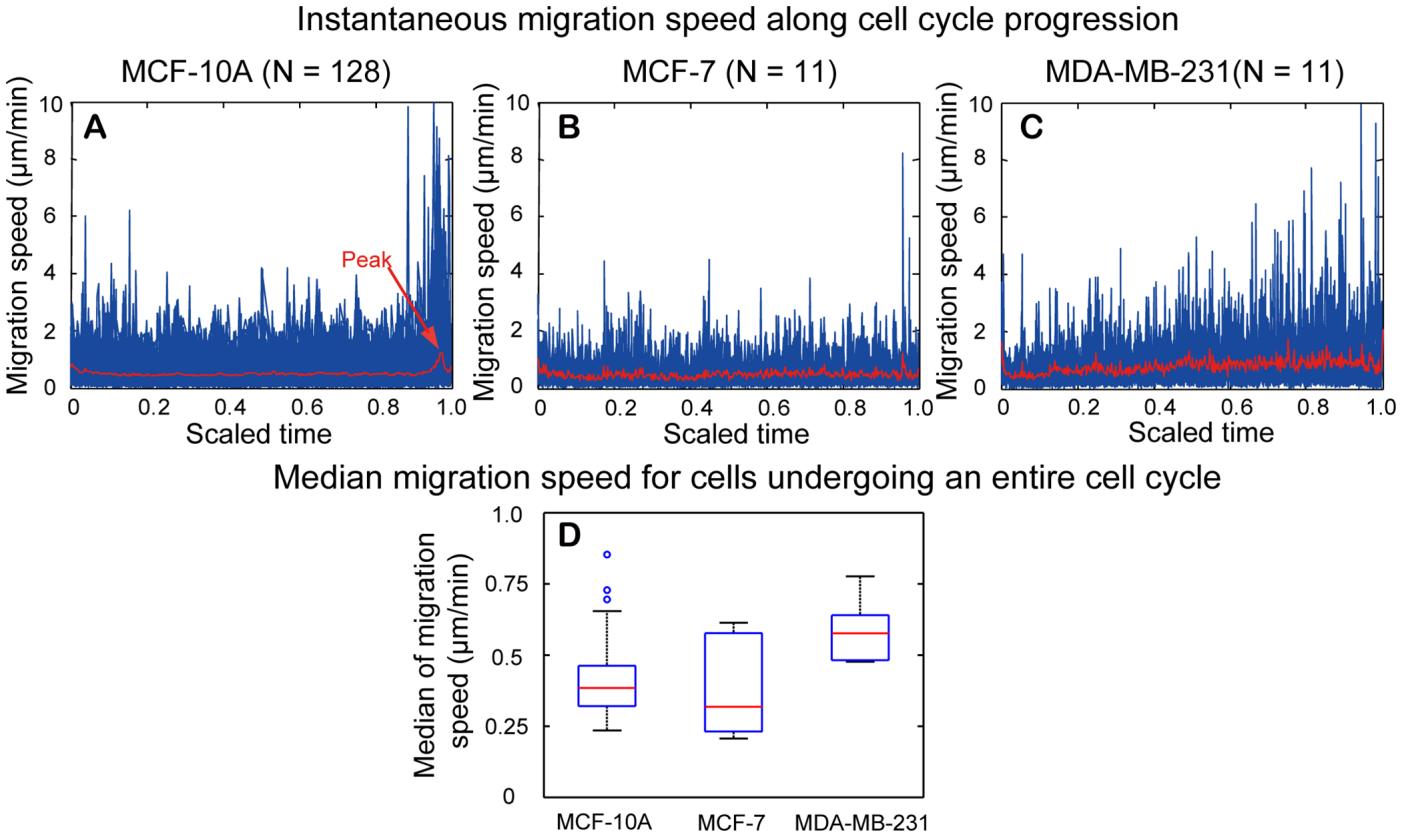
Instantaneous migration speed for cells undergoing an entire cell cycle. (A–C) Temporal change of instantaneous migration speed along cell cycle progression for MCF-10A, MCF-7, and MDA-MB-231 cells. The cell cycle was scaled to 0–1 to facilitate comparison among different cells within the same cell-line. Values of instantaneous migration speed for each cell examined (blue curves) were lined up with the scaled cell cycle and the mean value of all cells examined was shown by the red curve in each figure. (D) Boxplot of median instantaneous migration speed. MDA-MB-231 cells have highest median value, whereas MCF-10A and MCF-7 cells have comparable migration speed. The Wilcoxon rank rum test showed that there is statistically significant difference in distribution shift of median instantaneous migration speed between MCF-10A and MDA-MB-231, and between MDA-MB-231 and MCF-7 cells.

Temporal change in instantaneous migration speed along with the scaled cell cycle as well as the distribution of the median instantaneous migration speed for the three cell lines are shown in **Fig. 12**. For MCF-10A cells (**Fig. 12A**), a peak of mean value in instantaneous migration speed occurred before cell division, which reflects a rapid motion during the process of cell rounding before cell division. An increase in the mean value of instantaneous migration speed also occurred at the beginning of cell cycle, which marks the event of cytokinesis as the detection of cell division by our program occurred at telophase when cell bodies of two daughter cells became evident. Otherwise, mean values of instantaneous migration speed did not change much during interphase of cell cycle. For MCF-7 cells (**Fig. 12B**), the rapid increase of migration speed before cell division was less certain. The peak before cell division was mainly contributed by one outlier data with a small sample number investigated (N = 11). For MDA-MB-231 cells (**Fig. 12C**), the instantaneous migration speed increased modestly along cell cycle progression and the peak of migration speed appeared to occur at cell division. In reviewing the video, MDA-MB-231 cells indeed had unique abrupt motion during cell division.

The boxplots in **Fig. 12D** show the distribution of median values of the instantaneous migration speed for MCF-10A, MCF-7, MDA-MB-231 cell replicates. Note that MDA-MB-231 cells had the highest median value of instantaneous migration speed, 0.57 µm/min. The median values of instantaneous migration speed for MCF-10A and MCF-7 cells are 0.38 and 0.32 µm/min, respectively. Nonparametric Wilcoxon rank sum test indicated a statistically significant difference in distribution shift of median migration speed between MCF-10A and MDA-MB-231 cells, and between MDA-MB-231 and MCF-7 cells (**Table 2**).

#### b.2. Cell motion type

**Figure 13:**
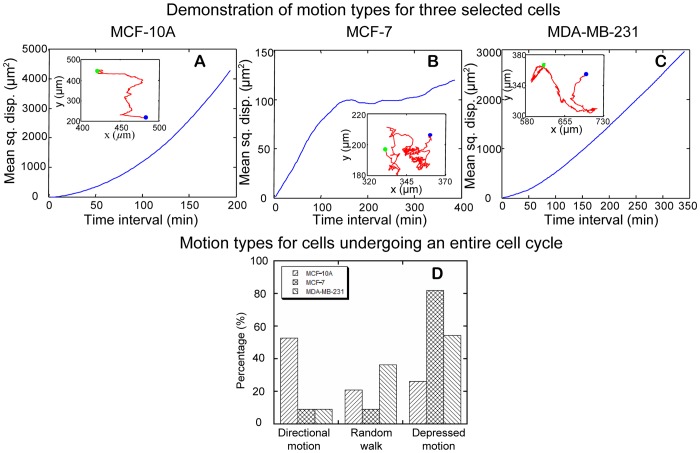
Motion types for cells undergoing an entire cell cycle. (A–C) show mean square displacement (MSD) as a function of time interval for a cell of MCF-10A, MCF-7, and MDA-MB-231, respectively. From left to right, the MSD curves show directional motion, depressed motion, and random walk, respectively. The inset in each figure is cell trajectories during the entire cell cycle for the selected cell. Blue and green dots represent the starting and ending points along cell trajectories, respectively. (D) Motion types for MCF-10A, MCF-7, and MDA-MB-231 cells undergoing an entire cell cycle. For MCF-10A cells (N = 128), over 50% of cells examined belong to directional motion. For MCF-7 cells (N = 11), over 80% of cells examined belong to depressed motion. For MDA-MB-231 cells (N = 11), 54.5% of cells examined are depressed motion. Fisher's Exact test indicates that motion types are significantly different among the three cell-lines (*p*-value  =  0.0001413).

**Fig**. **13A-C** shows three examples of MSD as a function of time interval for MCF-10A, MCF-7 and MDA-MB-231 cells, respectively. The insets are cell trajectories along their entire cell cycle. As defined in **Fig. 8C**, the three selected trajectories belong to directional motion, depressed motion, and random walk, respectively. A comparison of the motion type among the three cell-lines in the form of percentage is shown in **Fig. 13D**. More than 50% of MCF-10A cells had directional motion, 21% had random walk, and 26% had depressed motion. The depressed motion dominates the motion type for MCF-7 cells, as over 80% MCF-7 cells had depressed motion type. Similar to MCF-7 cells, less than 10% of MDA-MB-231 cells had directional motion. However, 36.3% of MDA-MB-231 cells had random walk and 54.5% had depressed motion type. Accordingly, both malignant human breast cancer cell lines rarely migrate with directional motion.

#### b.3. Cell motion range

Cell motion range is another parameter in evaluating cell migration. For a given trajectory, the maximum displacement between a point along a cell trajectory and the starting point of the trajectory is used to study cell motion range. In previous studies, motion range is mostly studied for all tracked cells, regardless of the variations in the length of tracking time period or different phases of cell cycle. In this study, the successful construction of cell lineage families makes it possible to compare cell motion range only for cells that underwent an entire cell cycle.

**Figure 14:**
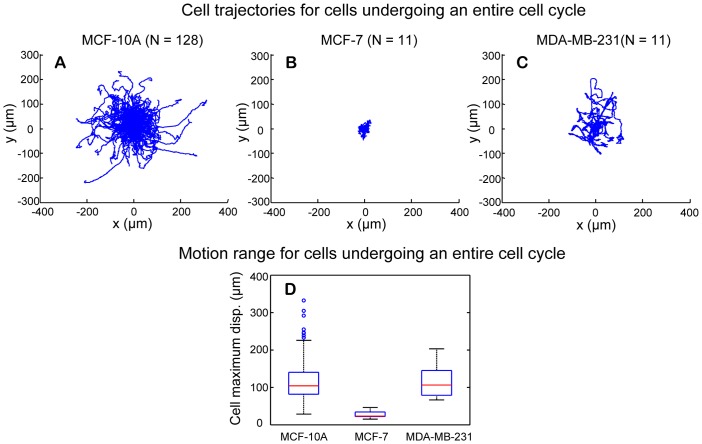
Motion range for cells undergoing an entire cell cycle. (A–C) The starting points of cell trajectories are shifted to the origin of the coordinate. MCF-10A and MDA-MB-231 cells show larger motion range than MCF-7 cells. (D) Boxplot of maximum displacement for the three cell-lines. MCF-10A and MDA-MB-231 cells have comparable mean values of their maximum displacement, whereas MCF-7 cells have much smaller mean value in maximum displacement. The Wilcoxon rank rum test showed that there is statistically significant difference in distribution shift of median maximum displacement between MCF-10A and MCF-7, and between MDA-MB-231 and MCF-7 cells.

**Fig. 14A, 14B, and 14C** show cell trajectories of cells that underwent an entire cell cycle for MCF-10A, MCF-7, and MDA-MB-231 cells, respectively. The starting point of each trajectory was shifted to the origin of the coordinate. One can see that MCF-7 cells had the smallest motion range and the motion range of MCF-10A cells was similar to that of MDA-MB-231 cells. The maximum displacement was calculated for each cell trajectory, and the boxplots of the median maximum displacement of cell trajectory for replicates of the three cell lines are shown in **Fig. 14D**. The medians of the maximum displacement for MCF-10A and MDA-MB-231 cells are 104 *µ*m and 106 *µ*m, respectively. The median of the maximum displacement for MCF-7 cells is much smaller, just 23 *µ*m. The *p* value of Wilcoxon rank sum test for distribution shift of median maximum displacement between MCF-10A and MDA-MB-231 was 0.8976, indicating that these two cell-lines have similar distribution of their median maximum displacement. In comparison, the *p* values for MCF-7 versus MCF-10A cells and for MCF-7 versus MDA-MB-231 cells are 6.549×10^-8^ and 2.835×10^-6^, respectively (**Table 2**). Thus, distribution of median maximum displacement in MCF-7 cells was significantly different from those in MCF-10A cells and MDA-MB-231 cells.

#### b.4. Migration direction related to the long axis of cells

**Figure 15:**
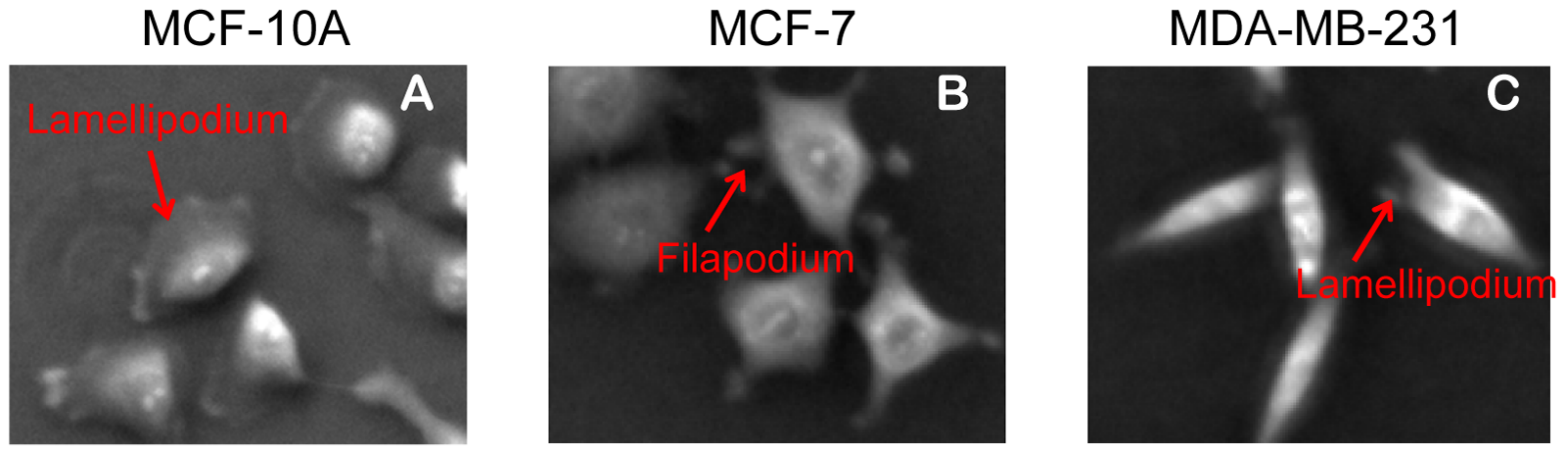
Negative phase contrast images of MCF-10A, MCF-7, and MDA-MB-231 cells. (A) MCF-10A cells have polarized protrusion structures of leading edge versus trailing edge. (B) MCF-7 cells have multiple protrusion structures around cell boundaries. (C) MDA-MB-231 cells have protrusion structures at the two ends of the long axes.

The different behaviors in cell migration of MCF-10A, MCF-7, and MDA-MB-231 cells can be related to cell shape and cell protrusion structures. As shown in **Fig. 15**, MCF-10A cells have well defined lamellipodial structures in the leading edge and minimum protrusion in the trailing edge, indicating less resistance from the trailing edge. Therefore, the leading edge dominates the cell migration direction and cells can achieve high migration speed. Note that lamellipodial structures for MCF-10A cells are generally along their long axis. MCF-7 cells have multiple protrusion structures around cell boundaries, such that these protrusion structures experience rapid retraction and extension without a dominant direction in cell migration. As a result, MCF-7 cells have limited migration range. MDA-MB-231 cells have the highest axis ratio among the three cell lines, and normally have protrusion structures at the two ends of the long axis. The leading and trailing edges may switch during migration, which results in back and forth locomotion.

**Figure 16:**
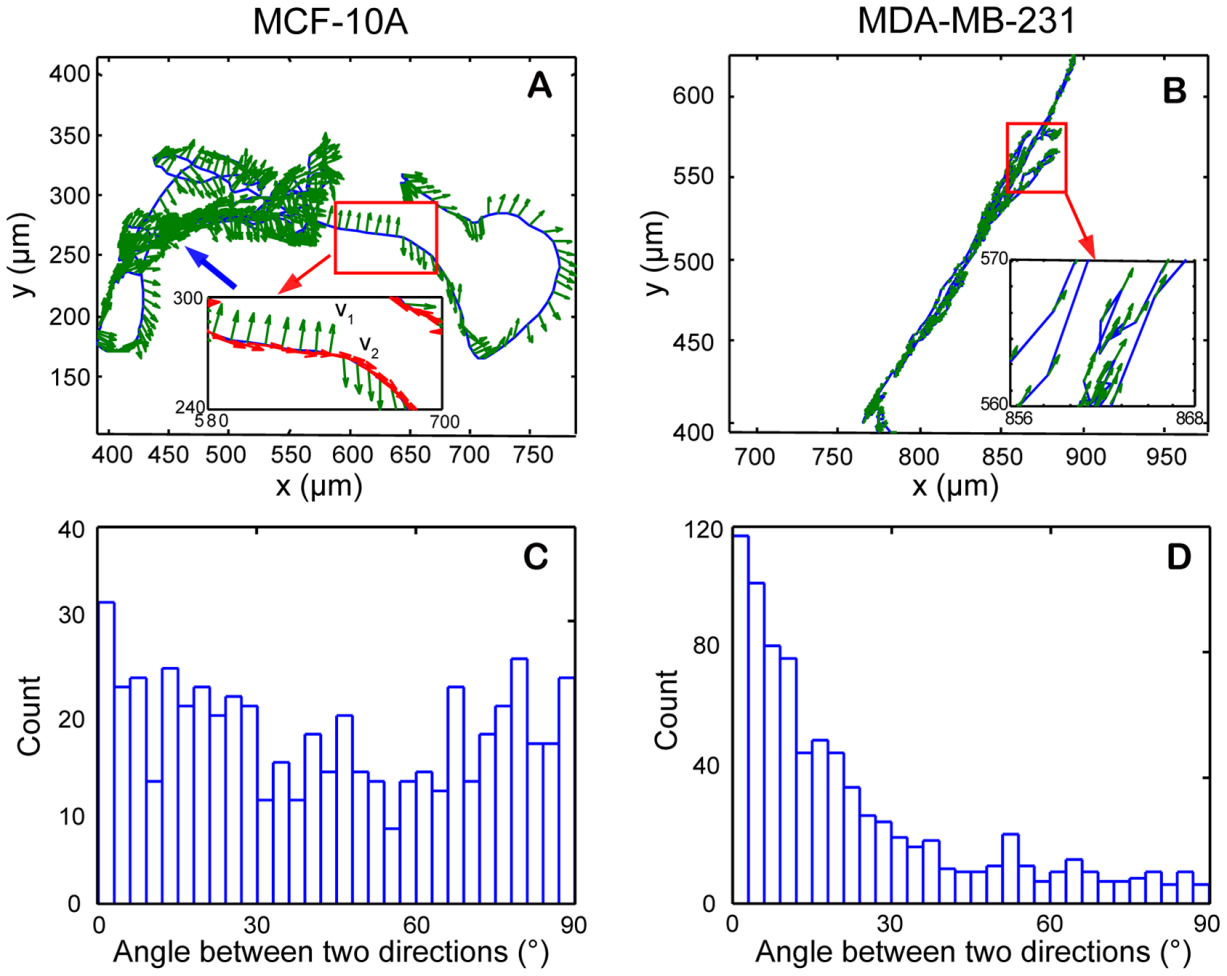
Correlation between direction of cell migration and cell long axis for MCF-10A and MDA-MB-231 cells. The direction of cell long axis along cell trajectories is shown with green arrows. (A) For the MCF-10A cell, the direction of cell long axis is mostly perpendicular to the direction of the cell trajectory. (B) For the MDA-MB-231 cell, the direction of cell long axis is mostly parallel to that of the cell trajectory. As shown in the inset of figure A, we used the unit vector ***v***_1_ and ***v***_2_ to indicate the direction of cell long axis and the direction of migration, respectively. (C, D) The histograms of the angle between the two unit vectors are shown for MCF-10A and MDA-MB-231 cells, respectively. The majority of data points for the MDA-MB-231 cell are close to zero degree, while the data points for the MCF-10A cell are distributed between 0 and 90 degree.

Since the direction of protrusion structure determines direction of cell motion, one could predict that the migration direction would be perpendicular to the long axis for MCF-10A cells, yet would be parallel to the long axis of MDA-MB-231 cells. Since neighboring cells can influence cell behavior, the correlation between direction of cell migration and cell long axis was investigated in an individual cell that was far apart from other cells. One MCF-10A and one MDA-MB-231 cell were selected for analysis when cell density in the field-of-the-view was low. **Fig. 16A and 16B** show the trajectories of the selected MCF-10A and MDA-MB-231 cell, respectively. The cell long axis direction along cell trajectories is shown with green arrows. For the MCF-10A cell (**Fig. 16A**), the direction of cell long axis is mostly perpendicular to the direction of the cell trajectory. For the MDA-MB-231 cell (**Fig. 16B**), the direction of cell long axis is mostly parallel to that of the cell trajectory. As shown in the inset of **Fig 16A**, we used the unit vector ***v***_1_ and ***v***_2_ to indicate the direction of cell long axis and the direction of migration, respectively. The correlation between direction of cell migration and cell long axis can be quantitatively studied by calculating the angle between the two unit vectors. **Fig. 16C and 16D** show the histograms of the angle between the two unit vectors for the selected MCF-10A cell and MDA-MB-231 cell, respectively. The majority of data points for the MDA-MB-231 cell are close to zero degree, while the data points for the MCF-10A cell are distributed between 0 and 90 degree. Similar results obtained from all MCF-10A and MDA-MB-231 cells undergoing an entire cell cycle are shown in **Fig. S2**. Although MCF-10A and MDA-MB-231 cells had comparable motion range, their migration directions, dictating by protrusion structures, are quite different.

## Conclusion

We have established a platform of automated live cell tracking system that includes a program to display tracking trajectories of specified cells frame by frame as well as a program for off-line editing to ensure high accuracy of cell tracking. This platform allows us to examine distributions of cell behaviors as well as temporal variations of cell body geometry and cell motion along with cell cycle progression for each individual cell that underwent an entire cell cycle. Examining cell behaviors of MCF-10A, MCF-7, and MDA-MB-231 cells with our established platform showed that (a) the highly metastatic MDA-MB-231 breast cancer cells had the greatest extent of heterogeneity in a 2D geometry distribution map and the highest median migration speed; (b) the non-invasive MCF-7 breast cancer cells had the largest median of cell body area and the smallest median of cell axis ratio. The large adhesion area to the 2D substrate along with the absence of polarization in cell body geometry are in agreement with low migration speed, predominant depressed motion type, and limited motion range found in MCF-7 cells; and (c) the non-malignant MCF-10A cells had similar median values in cell body area, cell axis ratio, and motion ranges with MDA-MB-231 cells. However, the median instantaneous migration speed of MCF-10A cells was significantly lower than that of MDA-MB-231 cells despite of its predominant directional motion type. Our finding that multiple cellular features are required to signify malignancy and metastatic potential for breast cancer cells are in agreement with a recent study reported that migration speed, migration directionality, together with spatiotemporal motion pattern are better indicative of metastatic potential of breast cancer cells [17]. Overall, the cell body geometry along with peripheral protrusion structures is closely associated with cell motion features.

To our knowledge, this is the first study to examine and compare temporal changes in cell geometry and migration speed along cell cycle progression among MCF-10A, MCF-7, and MDA-MB-231 cells. The false tracking rate of all data analyzed was greatly reduced by off-line editing. We noticed that the differences in cell geometry and cell motion among the three cell-lines studied were most evident during the mitosis phase (see **Fig 6**). Cells dividing or overlapping vertically were unique to both MCF-7 and MDA-MB-231 cancer cells but not found in MCF-10A cells, indicating this feature may be unique to malignant cancer cells. Abrupt changes in cell body geometry and cell motion were unique to MDA-MB-231 cells, indicating this feature may be indicative of metastatic potential. These novel observations will need to be verified in other breast cancer cells and further investigated in cancers of other tissue types.

The above phenomenon can be explained by (a) cytoskeleton dynamic plays an important role in cancer transformation and progression; and (b) cytoskeleton remodeling is most evident during mitosis. Accordingly, quantitative measurement of changes in cell geometry and cell motion during mitosis may be more sensitive to indicate cell malignancy and metastatic potential. It is now well established that physical and chemical inputs from microenvironment are jointly processed with intrinsic genetic lesions in tumor cells for invasion and metastasis [35]. Our automatic live cell tracking system can be applied to various cell culture systems with variations in extracellular matrix of desired chemical and physical properties, in micro fabrication of desired physical confinement, and in the presence or absence of cancer-associated fibroblasts, etc. to further uncover plasticity in cell geometry and cell motion that are unique to highly metastatic cancer cells.

Finally, cell-based live cell tracking with quantitative measurement of temporal variations in cell geometry and cell motion along with cell cycle progression will allow investigators to identify cellular features that are unique to distinct phase of cell cycle. Construction of cell lineage will allow investigators to identify cellular features that are passed from mother cells to daughter cells. Accordingly, our live cell tracking system will provide an invaluable tool to extract a wealth of information to better our understanding of many fundamental biological processes beyond cancer biology.

## Supporting Information

Figure S1 **False tracking event with time or number of cells in MCF-10A cells.** (A) The number of MCF-10A cells in the field-of-view increased with the time of tracking. (B) The number of false tracking event was negligible and sporadic before tracking time of 1500 min but was increased after tracking time of 1500 min. (C) The number of false tracking event was increased when the number of cells in the field-of-view went beyond 150.
(TIF)

Figure S2 **Angle between direction of cell migration and cell long axis for all cells undergoing an entire cell cycle.** (A) MCF-10 A cells (N = 128). (B) MDA-MB-231 cells (N = 11).
(TIF)

Video S1 **MCF-10A cells raw video without tracking.**
(AVI)

Video S2 **MCF-10A cells tracking video before offline editing correction.**
(AVI)

Video S3 **MCF-10A cells tracking video after offline editing.**
(AVI)
