# Supplementary figures and images for: Quantitative Characterization of Cell Behaviors through Cell Cycle Progression via Automated Cell Tracking
Source: PLoS One. 2014 Jun 9;9(6):e98762. doi: 10.1371/journal.pone.0098762 (PMC4049640; doi:10.1371/journal.pone.0098762)

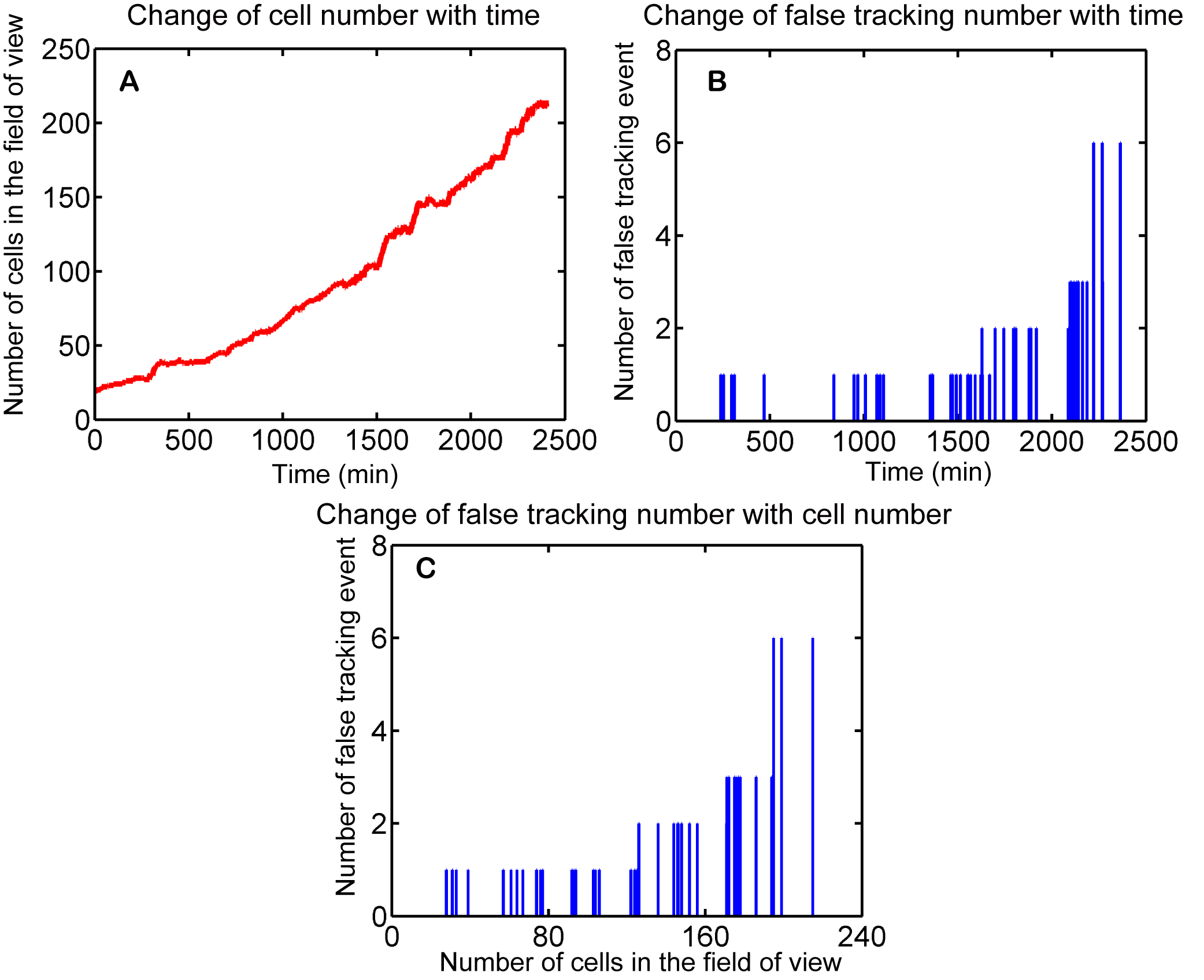

Supplement: Figure S1 — False tracking event with time or number of cells in MCF-10A cells. (A) The number of MCF-10A cells in the field-of-view increased with the time of tracking. (B) The number of false tracking event was negligible and sporadic before tracking time of 1500 min but was increased after tracking time of 1500 min. (C) The number of false tracking event was increased when the number of cells in the field-of-view went beyond 150. (TIF) [file pone.0098762.s001.tif]

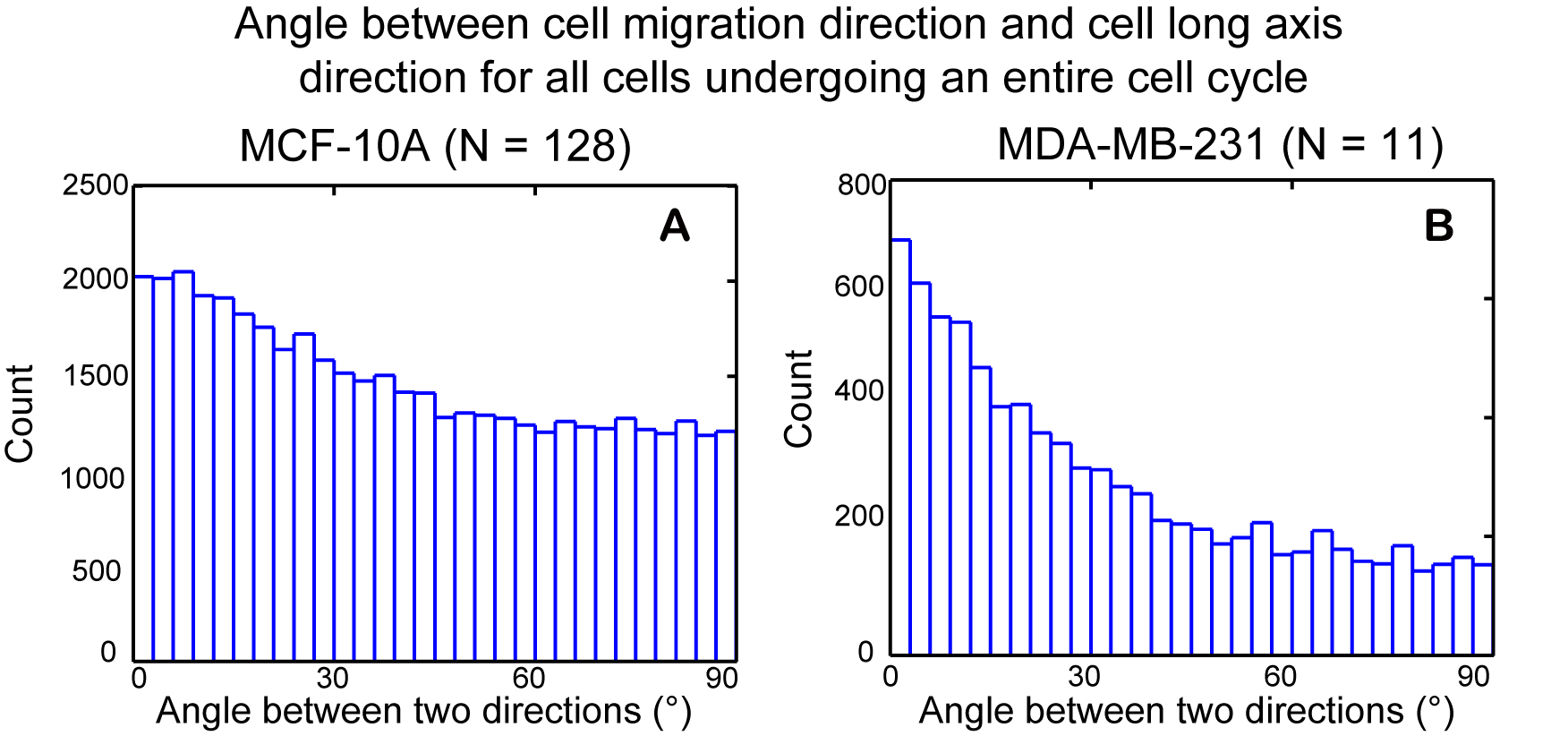

Supplement: Figure S2 — Angle between direction of cell migration and cell long axis for all cells undergoing an entire cell cycle. (A) MCF-10 A cells (N = 128). (B) MDA-MB-231 cells (N = 11). (TIF) [file pone.0098762.s002.tif]
